# TRPV1-dependent regulation of synaptic activity in the mouse dorsal motor nucleus of the vagus nerve

**DOI:** 10.3389/fnins.2013.00238

**Published:** 2013-12-13

**Authors:** Imran J. Anwar, Andrei V. Derbenev

**Affiliations:** ^1^Neuroscience Program, Tulane University, New OrleansLA, USA; ^2^Department of Physiology, Health Sciences Center, Tulane University, New OrleansLA, USA

**Keywords:** TRPV1, dorsal motor nucleus of the vagus, whole-cell patch-clamp recording, miniature postsynaptic currents

## Abstract

The dorsal motor nucleus of the vagus (DMV) is a key integrative point of the parasympathetic neuronal network localized in the dorsal vagal complex. Activity of neurons in the DMV is closely regulated by synaptic inputs, and regulation of excitatory and inhibitory synapsis by transient receptor potential vanilloid type 1 (TRPV1) has been demonstrated. Activation of TRPV1 by heat, protons, endovanilloids, endocannabinoids, and inflammatory mediators is well established. In our study we hypothesized that TRPV1 contributes to the synaptic transmission of DMV neurons at physiological range of temperature without additional stimuli. Using whole-cell patch-clamp recordings we evaluated the effect of a rapid increase of temperature on excitatory and inhibitory neurotransmission and the contribution of TRPV1 to this response. Rapid increase of temperature from 25 to 37°C increased the frequency of miniature excitatory post-synaptic currents (mEPSC) by 351.7%. The frequency of miniature inhibitory post-synaptic currents (mIPSC) also increased by 184.7%. 5′-iodoresiniferatoxin (5′-iRFT), a selective TRPV1 antagonist, prevented the increase of mEPSC and mIPSC frequency. In summary, our data demonstrate that at physiological range of temperature TRPV1 contributes to presynaptic neurotransmission of DMV neurons.

## Introduction

The dorsal vagal complex (DVC) is a main parasympathetic autonomic center. It encompasses the nucleus of the solitary tract (NTS), the dorsal motor nucleus of the vagus nerve (DMV), and the area postrema (AP) (Bailey, 2008). The NTS receives inputs from cranial visceral afferents that carry viscerosensory information. The interneurons in the NTS send outputs to various targets, including the DMV (Travagli et al., 1991; Travagli and Rogers, 2001; Davis et al., 2003, 2004; Glatzer and Smith, 2005). The DMV sends vagal projections to postganglionic neurons innervating subdiaphragmatic organs (Browning and Travagli, 2011), thereby regulating the function of the gastrointestinal tract, the cardiovascular system, and the respiratory system (Bauer et al., 2005; Shoudai et al., 2010; Cavanaugh et al., 2011; Zsombok et al., 2011b).

The transient receptor potential vanilloid type 1 (TRPV1) is a non-selective cation channel that displays high permeability to divalent cations such as Ca^2+^ and Mg^2+^ (Caterina et al., 1997; Tominaga et al., 1998; Venkatachalam and Montell, 2007). TRPV1 is activated by heat, protons, endovanilloids, endocannabinoids, and inflammatory mediators (Bevan and Yeats, 1991; Caterina et al., 1997; Tominaga et al., 1998; Zygmunt et al., 1999; Terenzi et al., 2013). In the peripheral nervous system, it has been shown that TRPV1 contributes to pain, thermosensation, chemosensation, and inflammatory responses (Bessac and Jordt, 2008; Gavva et al., 2008; Patwardhan et al., 2010). While the contribution of TRPV1 to physiological responses has been thoroughly investigated in the peripheral nervous system, less information is known about TRPV1-dependent regulation of synaptic transmission in the central nervous system (CNS). TRPV1 expression in the CNS is restricted to specific areas. TRPV1 is expressed in the cerebral cortex, the hypothalamus, the brainstem and the hindbrain, as shown by different methods (Cristino et al., 2006; Derbenev et al., 2006; Pingle et al., 2007; Cavanaugh et al., 2011; Zsombok et al., 2011b; Gao et al., 2012).

Previous studies demonstrated the functional properties of TRPV1 in the DVC (Derbenev et al., 2006; Peters et al., 2010, 2011; Shoudai et al., 2010; Cavanaugh et al., 2011; Fawley et al., 2011; Zsombok et al., 2011b). TRPV1 drives synaptic activity of cranial visceral afferents providing continuous inputs to the NTS independently of afferent activity (Peters et al., 2010; Shoudai et al., 2010). Furthermore, TRPV1 enhances neurotransmitters release to DMV neurons. Activation of TRPV1 by capsaicin, an exogenous agonist, produces a robust increase of both miniature excitatory postsynaptic current (mEPSC) frequency and miniature inhibitory postsynaptic current (mIPSC) frequency in DMV neurons demonstrating that TRPV1 activation increases synaptic activity in the DVC (Derbenev et al., 2006).

Thermal activation of TRPV1 has also been demonstrated. TRPV1 has a heat activation threshold of ~43°C *in vitro* (Caterina et al., 1997; Tominaga et al., 1998; Premkumar and Ahern, 2000; Gavva et al., 2007; Grandl et al., 2010; Shoudai et al., 2010). Due to the polymodal characteristics of TRPV1, the heat activation threshold can be reduced by a variety of processes, including PKC phosphorylation, proton activation and repeated exposure to heat (Ji et al., 2002; Moriyama et al., 2005; Jay, 2007). Lowering the heat activation threshold could allow TRPV1 to be potentiated at physiological temperatures (~37°C). Based on the above-mentioned observations, we hypothesized that TRPV1 is active at physiological range of temperatures and enhances synaptic activity to DMV neurons. To test this, we conducted patch-clamp recordings where the temperature was increased from 25 to 37°C to demonstrate thermal activation of TRPV1 in the DMV. Our data revealed that at 37°C, the frequency of mEPSCs and the frequency of mIPSCs increased as compared to recordings conducted at 25°C. Upon further examination, we found that the potentiation of excitatory and inhibitory neurotransmission to DMV neurons was a result of thermal activation of presynaptic TRPV1 receptors. Our results indicate that TRPV1 regulates synaptic inputs to DMV neurons at physiological temperatures.

## Materials and methods

Experiments were performed on male CD1 mice (7–8 weeks old; Harlan) following the National Institutes of Health Guide for the Care and Use of Laboratory Animals and were approved by Tulane University's Institutional Animal Care and Use Committee.

### Brainstem slice preparation

Transverse brainstem slices were prepared from male mice as described previously (Zsombok et al., 2011a). Mice were deeply anesthetized by isoflurane inhalation and sacrificed by decapitation while anesthetized. Brains were rapidly removed and immersed in ice-cold (0–4°C) oxygenated artificial cerebrospinal fluid (ACSF) containing the following: 124 mM NaCl, 3 mM KCl, 26 mM NaHCO_3_, 1.4 mM NaH_2_PO_4_, 11 mM glucose, 1.3 mM CaCl_2_, and, 1.3 mM MgCl_2_. The pH was adjusted to physiological ranges (7.3–7.4), with an osmolality of 290–310 mOsm/kg. Transverse brainstem slices (300 μm) containing the DMV were made using a vibrating microtome (Vibratome Series 1000; Technical Products). Slices were maintained in an oxygenated bath solution at 35°C for at least 1 h before performing experiments. Slices were then transferred to a recording chamber mounted on a fixed stage under an upright microscope (Nikon FN1).

### Whole-cell patch-clamp recordings

DMV neurons were visually identified in coronal brainstem slices and were patch-clamped with a glass pipette with series resistance between 2 to 4 MΩ. The electrodes were filled with a solution containing the following: 130 mM Cs^+^-gluconate, 1 mM NaCl, 5 mM EGTA, 10 mM HEPES, 1 mM MgCl_2_, 1 mM CaCl_2_, 3 mM CsOH, 2-4 mM Mg-ATP, buffered to pH = 7.3–7.4 (with CsOH). Electrophysiological signals were low-pass filtered at 2–5 kHz, digitized at 88 kHz, recorded using an Axopatch 700 B amplifier (Molecular Devices). Excitatory post-synaptic currents (EPSCs) were examined at a holding potential of −60 mV while inhibitory post-synaptic currents (IPSCs) were recorded at a holding potential of 0 mV.

### Temperature setting and protocol

The temperature was monitored by an extracellular probe placed in the chamber 1 cm apart from the slice. The probe was placed upstream from the slice, close to where the ACSF entered the chamber. Therefore the temperature changes reflected by the probe occurred with a slight delay at the level of the brain slice. A Koolance device (Warner instrument) was used to heat the ACSF entering the chamber, allowing for the precise control of the temperature inside the chamber. In control conditions, DMV neurons were maintained at 25°C. To investigate the temperature-dependent contribution of TRPV1 to the synaptic transmission, recordings were made while the ACSF was gradually heated up to 37°C. Then, the temperature was decreased back to 25°C.

### Drug application

Recordings were performed with tetrodotoxin (1 μM; TTX; Tocris Bioscience) in ACSF to block action potentials and monitor mEPSCs or mIPSCs. In addition, the TRPV1 antagonist 5′-iodoresiniferatoxin (1 μM; 5′-iRFT; Tocris Bioscience) was dissolved in ethanol and diluted in ACSF (final concentration of ethanol <0.01% by volume).

### Data analysis

The recordings were analyzed with pClamp 10 software (Molecular Devices). Miniature IPSCs and EPSCs were analyzed offline using MiniAnalysis (Synaptosoft). The effects of temperature on mEPSCs and mIPSCs were analyzed within individual cells using the Kolmogorov-Smirnov test. The effects of temperature across neuron groups were analyzed using a paired two-tailed Student's t test. Values are expressed as means ± s.e.m.

## Results

### TRPV1 potentiates excitatory neurotransmission to DMV neurons in temperature-dependent manner

Patch-clamp recordings from NTS neurons demonstrated that increase of temperature potentiates excitatory neurotransmission in a TRPV1-dependent manner (Peters et al., 2010; Shoudai et al., 2010). To reveal the contribution of TRPV1 to temperature-dependent neurotransmission in the DMV, we used patch-clamp whole-cell recordings. The frequency and amplitude of mEPSCs were examined at −60 mV using Cs-gluconate solution in the recording pipettes to block K^+^ currents (Bach and Smith, 2012). In control condition, the temperature of ACSF was maintained at 25°C. Then, the ACSF was heated up to 37°C within 5 min. A schematic illustration of the temperature protocol is depicted on Figures 2B, 4B.

**Figure 1:**
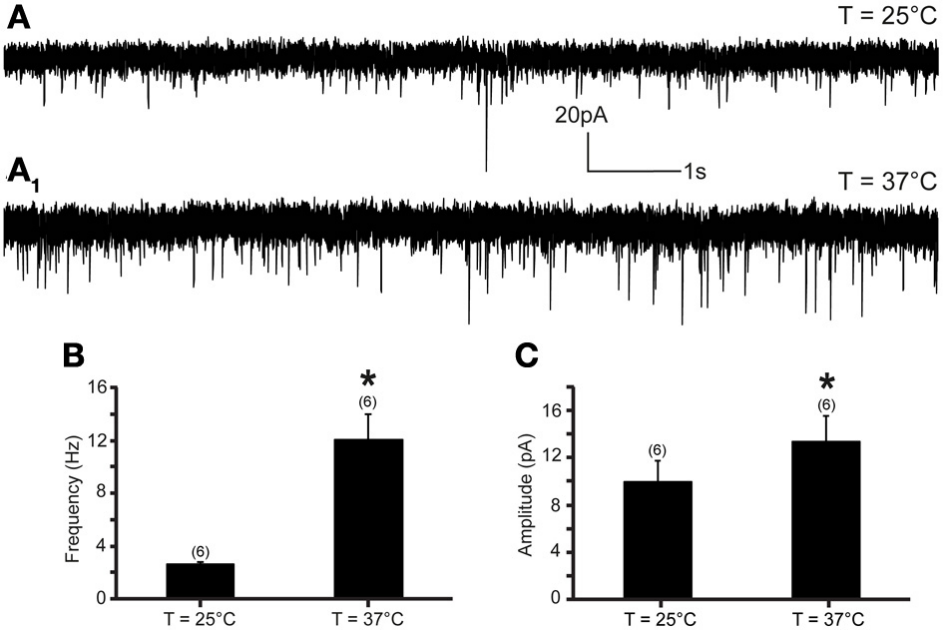
**Excitatory neurotransmission to DMV neurons is potentiated by TRPV1 at physiological temperatures. (A)** Continuous recording of mEPSCs at 25°C **(A)** and after elevation of temperature from 25 to 37°C **(A_1_)** in TTX (1 μM). **(B,C)**. Bar graph summarizing the effect of temperature on mEPSC frequency **(B)** and mEPSC amplitude **(C)**. ^*^Significance (*p* < 0.05).

**Figure 2:**
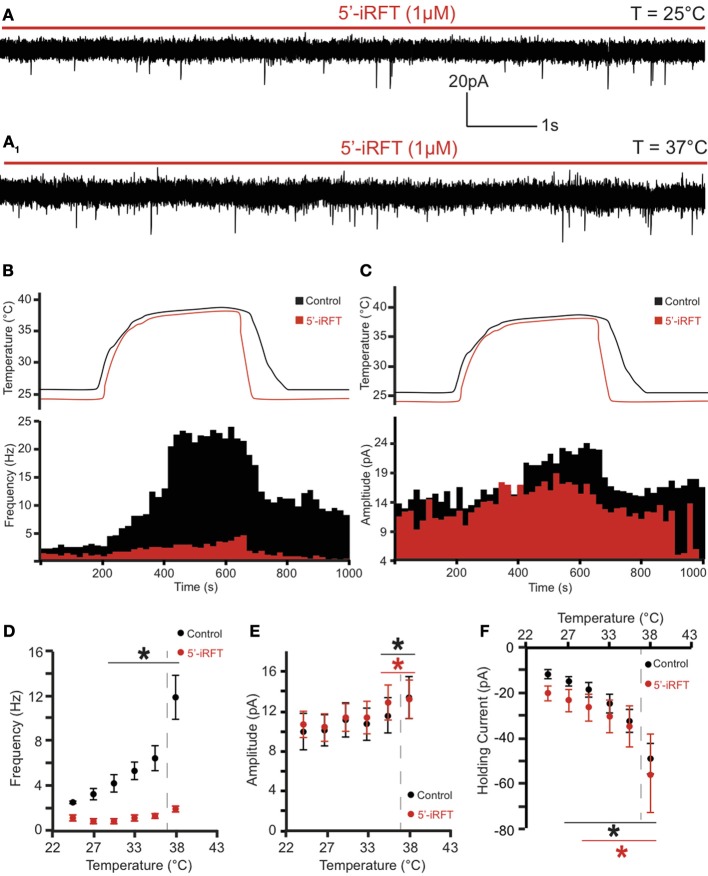
**TRPV1 antagonist prevents the potentiation of excitatory neurotransmission caused by TRPV1 activation to DMV neurons. (A)** Continuous recording of mEPSCs in the presence of 5′-iRFT (1μM) and TTX (1μM) at 25°C **(A)** and after increase of temperature from 25°C to 37°C **(A_1_)**. **(B,C)**. The effect of TRPV1 antagonist on the increase of mEPSC frequency **(B)** and amplitude **(C)**. **(D–F)**. Summary for temperature response on mEPSC frequency **(D)**, mEPSC amplitude **(E)**, and total inward current **(F)** (*n* = 6). Black traces indicate control conditions. Red traces indicate recordings conducted in the presence of 5'-iRFT, a TRPV1 antagonist. Dashed-line indicates physiological temperature. ^*^Significance (*p* < 0.05).

The increase of temperature from 25 to 37°C produced a significant increase in mEPSC frequency and amplitude in all recorded neurons (Figure 1). At 25°C, the average mEPSC frequency was 2.64 ± 0.1 events per second (range from 2.15 to 2.98 events per second; *n* = 6). Rapid increase of temperature to 37°C triggered a 351.7% increase of mEPSCs frequency to 12.02 ± 1.7 events per second (range from 4.34 to 17.95 events per second; *n* = 6; *P* < 0.05) (Figure 1B). The temperature-induced changes were rapid, reversible and did not diminish overtime in any of the recorded cells (*n* = 6) (Figure 2B).

To test whether the temperature-dependent increase of mEPSC frequency was due to TRPV1 activation, 5′-iRFT, a selective TRPV1 antagonist was added to the ACSF for 30 min before recordings were performed. Application of 5′-iRFT (1 μM) prevented the increase of mEPSC frequency triggered by temperature elevation (Figures 2A,B,D). In the presence of 5′-iRFT the average mEPSC frequency was 1.24 ± 0.21 events per second (range from 0.96 to 1.88 events per second; *n* = 6) (Figure 2D, red trace). Rapid increase of temperature to 37°C in the presence of 5′-iRFT prevented the increase of mEPSC frequency. The average mEPSC frequency was 2.20 ± 0.26 events per second at 37°C (range from 1.51 to 2.96 events per second; *n* = 6; *P* > 0.05) (Figure 2D), which was not significantly different as compared to mEPSC frequency measured at 25°C. Our results suggest that the temperature-dependent potentiation of excitatory neurotransmission is driven by activation of TRPV1.

In addition, we assessed the effect of the rapid increase of temperature on mEPSC amplitude. We found that an increase of temperature from 25 to 37°C resulted in a significant increase of mEPSC amplitude (Figures 1C, 2C). At 25°C the average mEPSC amplitude was 10.47 ± 1.6 pA (range from 7.25 to 14.97 pA; *n* = 6). Rapid increase of temperature to 37°C significantly increased the amplitude of mEPSCs in all recorded DMV neurons. The average mEPSC amplitude was 13.88 ± 1.8 pA (range from 8.73 to 19.09 pA; *n* = 6; *P* < 0.05) in ACSF at 37°C (Figure 1C). To determine if the temperature-dependent increase of amplitude is TRPV1 driven, amplitudes were compared in the presence and absence of 5-iRFT. Application of TRPV1 antagonist, 5′-iRFT, did not block the increase of mEPSC amplitude in response to temperature increase (Figures 2C,E, red trace). Failure to prevent the increase of mEPSC amplitude with a TRPV1 antagonist suggests that the effect occurs in a TRPV1-independent fashion.

In addition, increase of temperature to 37°C modulated mEPSC kinetics. Rise-time and decay time were both significantly reduced at 37°C (Table 1). The change in the mEPSC kinetics was not blocked by 5-iRFT application, suggesting that the effect also occurs in a TRPV1-independent fashion.

**Table 1:** **Rise time and decay time response to temperature changes in DMV neurons in control condition and in the presence of 5-iRFT**.

		**T = 25°C**		**T = 37°C**	
	***n Cells***	**Mean**	**Range**	**Mean**	**Range**
**mEPSCs**					
Rise time (ms)	6	1.76 ± 0.4	1.07–2.73	1.07 ± 0.3^*^	0.56–2.30
Decay time (ms)	6	4.23 ± 1.0	1.98–8.89	2.54 ± 0.8^*^	1.14–6.71
**in 5′-iRFT**					
Rise time (ms)	6	1.02 ± 0.2	0.60–1.71	0.80 ± 0.2^*^	0.42–1.71
Decay time (ms)	6	1.85 ± 0.2	1.47–2.73	1.37 ± 0.1^*^	1.03–1.77
**mIPSCs**					
Rise time (ms)	7	1.83 ± 0.2	0.91–2.07	0.89 ± 0.1^*^	0.65–1.59
Decay time (ms)	7	6.82 ± 0.3	5.88–7.91	2.37 ± 0.3^*^	1.39–2.72
**in 5′-iRFT**					
Rise time (ms)	6	2.57 ± 0.3	1.09–4.30	1.80 ± 0.2^*^	0.88–2.58
Decay time (ms)	6	10.74 ± 0.5	8.20–12.37	5.49 ± 0.5^*^	3.55–7.87

* Significance (*P* < 0.05)

Temperature-induced currents were also investigated. Rapid increase of temperature produced a significant shift of the holding current. Rapid increase of temperature from 25 to 37°C shifted the holding current from −11.87 ± 1.9 pA (range −6.12 to −18.53) to −49.21 ± 6.7 pA (range −28.04 to −76.49; *n* = 6; *P* < 0.05) (Figure 2F). Application of 5′-iRFT did not block the inward shift in holding current (Figure 2F, red trace).

Our results suggest that rapid increase of temperature from 25 to 37°C had multiple effects, but potentiation of presynaptic release of excitatory neurotransmitters to DMV neurons is TRPV1-dependent.

### TRPV1 potentiates inhibitory neurotransmission to DMV neurons in a temperature-dependent manner

**Figure 3:**
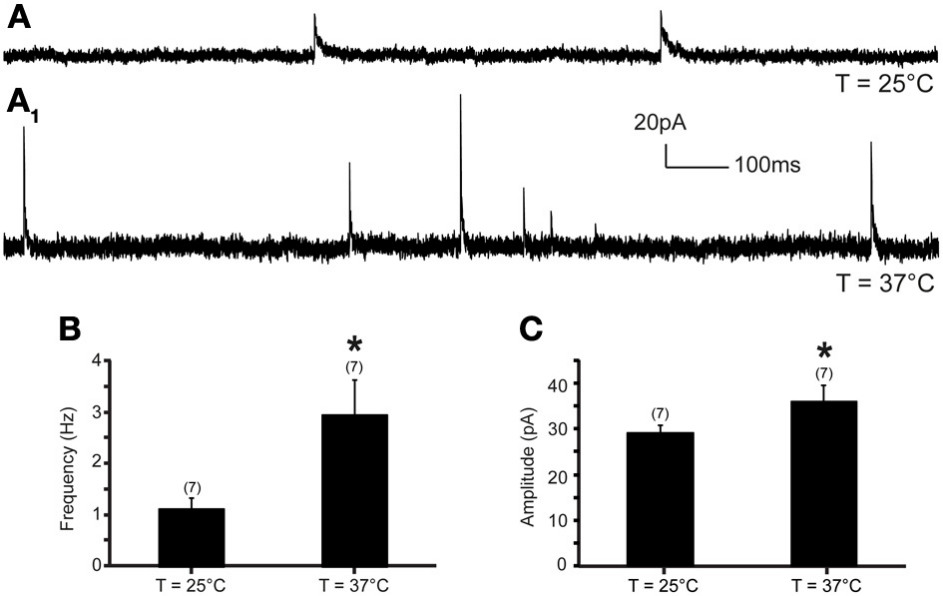
**Inhibitory neurotransmission to DMV neurons is potentiated by TRPV1 at physiological temperatures. (A)**. Continuous recording of mIPSCs at 25°C **(A)** and after increase of temperature from 25 to 37°C **(A_1_)** in TTX (1 μM). **(B,C)**. Bar graph summarizing the effect of temperature on mIPSC frequency **(B)** and mIPSC amplitude **(C)**. ^*^Significance (*p* < 0.05).

**Figure 4:**
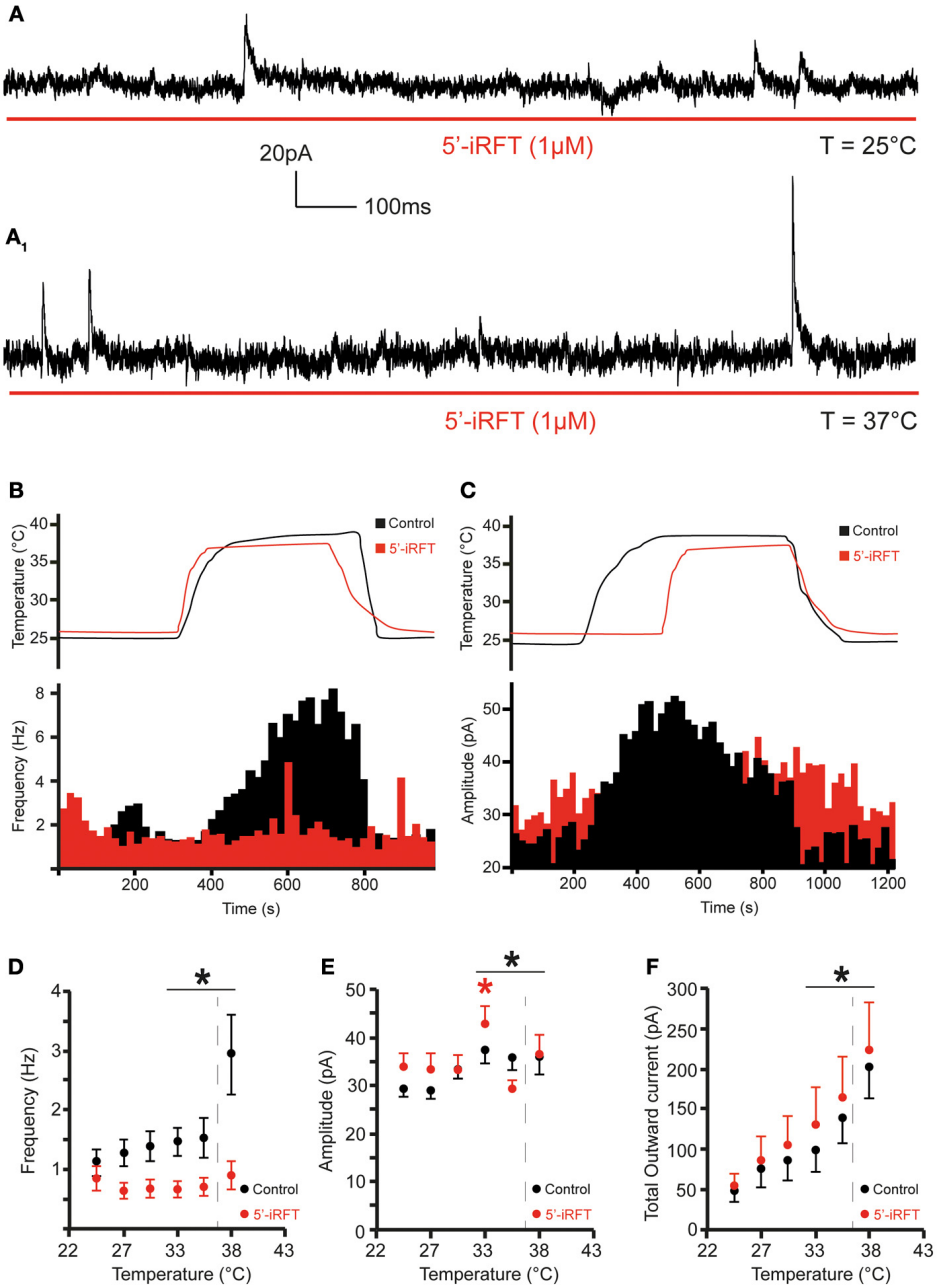
**TRPV1 antagonist prevents the increase of inhibitory neurotransmission to DMV neurons caused by TRPV1 activation. (A)**. Continuous recording of mIPSCs in the presence of 5′-iRFT (1 μM) and TTX (1 μM) at 25°C **(A)** and after rise of temperature from 25 to 37°C **(A_1_)**. **(B,C)**. The effect of TRPV1 antagonist on the increase of mIPSC frequency **(B)** and amplitude **(C)** (red traces are recordings with 5′-iRFT). **(D–F)**. Summary for temperature response on mIPSC frequency **(D)**, mIPSC amplitude **(E)**, and total outward current **(F)** (*n* = 7). Black traces indicate control conditions. Red traces indicate recordings conducted in the presence of 5′-iRFT, a TRPV1 antagonist. Dashed-line indicates physiological temperature. ^*^Significance (*p* < 0.05).

The frequency and amplitude of mIPSCs were examined at 0 mV. Increase of temperature from 25 to 37°C produced a significant increase of mIPSC frequency and amplitude in all recorded neurons (Figure 3). At 25°C, the average mIPSC frequency was 1.10 ± 0.2 events per second (range from 0.27 to 1.93 events per second; *n* = 7). Rapid increase of temperature to 37°C triggered a 184.7% increase of mIPSC frequency to 2.93 ± 0.7 events per second (range from 0.63 to 6.11 events per second; *n* = 7; *P* < 0.05) (Figure 3B). The temperature-induced changes were rapid, reversible and did not diminish overtime (*n* = 7) (Figure 4B). To test whether the increase of mIPSC frequency was caused by TRPV1 activation, mIPSCs were recorded in the presence of 5′-iRFT, a TRPV1 antagonist. Application of 5′-iRFT (1 μM) prevented the increase of mIPSC frequency triggered by temperature elevation (Figures 4A,B,D). In the presence of 5′-iRFT, the mean mIPSC frequency was 0.86 ± 0.26 events per second (range from 0.20 to 1.77 events per second; *n* = 6). After rapid increase of temperature to 37°C in ACSF containing 5′-iRFT, we observed no significant changes in mIPSC frequency. The average mIPSC frequency was 0.72 ± 0.25 events per second (range from 0.35 to 1.89 events per second; *n* = 6; *P* > 0.05) (Figure 4D). Our results suggest that the temperature-dependent potentiation of inhibitory neurotransmission is driven by activation of TRPV1.

Next, we assessed the effect of temperature elevation on mIPSC amplitude. We found that rapid increase of temperature from 25 to 37°C significantly increased the amplitude of mIPSCs (Figures 3C, 4C). At 25°C, the mean mIPSC amplitude was 29.18 ± 1.5 pA (range from 24.54 to 35.75 pA, *n* = 7). Rapid increase of temperature to 37°C significantly increased amplitude of mIPSCs in all recorded DMV neurons. The average amplitude was increased to 35.84 ± 3.6 pA (range from 24.46 to 50.11 pA; *n* = 7; *P* < 0.05) at 37°C (Figure 3C). Application of the TRPV1 antagonist, 5′-iRFT, did not block the increase of mIPSC amplitude in response to temperature increase (Figures 4C,E, red trace). Failure to block the increase of mIPSC amplitude with a TRPV1 antagonist suggests that the effect occurs in a TRPV1-independent fashion.

In addition, increase of temperature to 37°C modulated mIPSC kinetics. Rise-time and decay time were both significantly reduced at 37°C (Table 1). The change in mIPSC kinetics was not blocked by 5-iRFT application, suggesting that the effect also occurs in a TRPV1-independent fashion.

Finally, temperature-induced currents were investigated. Rapid increase of temperature produced a significant shift of the holding current. Rapid increase of temperature from 25 to 37°C shifted the holding current from 49.32 ± 13.5 pA at 25°C to 202.3 ± 38.3 pA at 37°C (*n* = 7; *P* < 0.05) (Figure 4F). Application of 5′-iRFT did not block the outward shift in holding current (Figure 4F, red trace).

Our results suggest that rapid increase of temperature form 25 to 37°C potentiate presynaptic release of inhibitory neurotransmitters to DMV neurons in TRPV1-dependent manner.

## Discussion

The results of our study provide novel information about TRPV1-dependent regulation of excitatory and inhibitory neurotransmission in the DVC. Our data demonstrate that TRPV1 modulates synaptic transmission of DMV neurons in two different ways. First, at 37°C, mEPSC frequency is increased due to presynaptic activation of TRPV1. Second, mIPSC frequency is increased due to presynaptic activation of TRPV1. Our results show that TRPV1 is involved in the regulation of both excitatory and inhibitory neurotransmission to DMV neurons. Physiological temperatures, in our case 37°C, activate presynaptic TRPV1, thus increasing excitatory and inhibitory neurotransmitters release to DMV neurons.

It has been shown previously that activation of TRPV1 by capsaicin resulted in increased mIPSC and mEPSC frequency in DMV neurons (Derbenev et al., 2006). TRPV1 is localized on both excitatory and inhibitory presynaptic terminals synapsing with DMV neurons and TRPV1 activation produces an increase of neurotransmitter release from these presynaptic terminals. Also, it has been shown that TRPV1 drives asynchronous synaptic activity to the NTS independently of afferent activity (Peters et al., 2010; Shoudai et al., 2010). Here we demonstrated that physiological range of temperature (37°C) alone drives frequency of mEPSCs and mIPSCs in TRPV1-dependent manner.

It has been reported that TRPV1 has a thermal threshold of 43°C *in vitro* (Caterina et al., 1997; Tominaga et al., 1998; Premkumar and Ahern, 2000; Gavva et al., 2007; Yao et al., 2010). The heat-induced activation of TRPV1 is caused by a negative shift of the voltage dependence of activation. At 0 mV and 25°C, the probability of opening is ~0.10 while at 35°C, the probability of opening is ~0.40. At −60 mV, probability of opening stays low, reaching ~0.10 at 35°C (Voets et al., 2004). Protons and PKC sensitize TRPV1 to lower temperatures by increasing the negative shift of voltage dependence of activation (Premkumar et al., 2002; Voets et al., 2004). Seeing the robust activation of TRPV1 even at −60 mV, it is plausible that TRPV1 is potentiated in the DVC. Clearly, further studies are needed to elucidate the exact mechanisms of sensitization of TRPV1 in the DVC.

5′-iRFT application was used to block TRPV1 in some of our recordings. Previously, it has been shown that in control conditions, 5′-iRFT does not effect either excitatory or inhibitory inputs to DMV neurons at 1 μM concentration (Zsombok et al., 2011a). In contrast, our data show that 5′-iRFT significantly reduced mEPSC frequency: mESPC frequency was 2.64 ± 0.1 events per second in the control group (range from 2.15 to 2.98 events per second; *n* = 6) and 1.24 ± 0.21 events per second in the 5′-iRFT group (range from 0.96 to 1.88 events per second; *n* = 6; *P* < 0.05). Our differential findings could be explained by the fact that the 5′-iRFT effect was investigated across group and not across individual neurons: the difference of basal frequency between the control group and the 5′-iRFT group is likely due to variability between the neurons recorded in each group. Even though the basal mEPSC frequency is lower in the 5′-iRFT group, we would expect similar temperature-dependent increase of mEPSC frequency if 5′-iRFT were not present in the ACSF.

Our data also indicate that a rapid increase of temperature results in an increase of mPSC amplitude in a linear fashion, and 5′-iRFT failed to block this increase (Figures 2E, 4E) suggesting that temperature-induced increase of mPSC amplitude is TRPV1 independent. Furthermore, mPSC kinetics were influenced by temperature in the same linear-fashion. Decay-time and rise-time were significantly reduced at 37°C compared to 25°C (Table 1). As the temperature increased, the 10–90% rise time and decay time constant of mIPSC and mEPSC decreased. This is consistent with previous studies demonstrating relationship between temperature and PSC kinetics (Taschenberger and Gersdorff von, 2000; Wall et al., 2002; Kushmerick et al., 2006; Postlethwaite et al., 2007). Temperature increase from 25 to 37°C accelerated mEPSC kinetics and increased mEPSC amplitude in the calyx of Held due to a temperature-dependent scaling of reaction rate constant of AMPA receptors. Specifically, the changes were caused by accelerated agonist binding, unbinding and kinetics of AMPA receptors (Postlethwaite et al., 2007). We can speculate that the same scaling of reactions rates might occur in the DMV due to increase of temperature.

It is well established that DMV neurons receive synaptic inputs from the NTS and provide tonic inputs to visceral organs (Travagli and Rogers, 2001). Also, DMV neurons exhibit slow, spontaneous, pacemaker-like activity by tonically firing action potentials (Barrett et al., 2006; Travagli et al., 2006; Browning and Travagli, 2011). Microinjections of glutamate or GABA receptor antagonists into the DVC confirmed that inhibitory inputs play a significant role in regulating the rate of the pacemaker-like activity of DMV neurons, while the excitatory inputs were not involved (Browning and Travagli, 2011). This suggests that the NTS provides tonic GABAergic inputs to the DMV to regulate its activity, while excitatory inputs to the DMV have little effect on the activity of the DMV. Our study suggests that both the excitatory and inhibitory inputs are tonically potentiated at physiological temperature due to the presence of presynaptic TRPV1. The presence of TRPV1 on GABAergic terminals could thus constitute an additional regulatory mechanism of the vagal tone. Furthermore, tonic TRPV1 activation could reduce the pacemaker-like activity of DMV neurons and therefore reduce the motor vagal output.

Our study revealed that TRPV1 tonically drives excitatory and inhibitory inputs to the DMV at physiological temperatures. These findings provide new insights into TRPV1 function in the CNS and the autonomic nervous system. While TRPV1 serves primarily as a noxious stimuli integrator in the peripheral nervous system, our study suggests that TRPV1 exerts different functions in the CNS. At physiological temperatures, TRPV1 plays a novel role in the neurotransmission of the DMV, and therefore also contributes to the vagal motor output and the control of visceral organs.

## Author contributions

Imran J. Anwar performed experiments; Imran J. Anwar analyzed data; Imran J. Anwar and Andrei V. Derbenev interpreted results of experiments; Imran J. Anwar prepared figures; Imran J. Anwar and Andrei V. Derbenev edited and revised manuscript; Imran J. Anwar and Andrei V. Derbenev approved final version of manuscript; Andrei V. Derbenev conception and design of research; Imran J. Anwar and Andrei V. Derbenev drafted manuscript.

### Conflict of interest statement

The authors declare that the research was conducted in the absence of any commercial or financial relationships that could be construed as a potential conflict of interest.
